# Comparative Pharmacokinetic Profiles of Puerarin in Rat Plasma by UHPLC-MS/MS after Oral Administration of *Pueraria lobata* Extract and Pure Puerarin

**DOI:** 10.1155/2020/4258156

**Published:** 2020-04-14

**Authors:** Guozhe Zhang, Jianwei Ji, Mingzhong Sun, Yuqiao Ji, Hongjian Ji

**Affiliations:** ^1^Department of Translational Medicine, Jiangsu Vocational College of Medicine, 283 South of Republic Road, Yancheng 224005, China; ^2^Department of Pharmacy, Yancheng Third People's Hospital, 2 West of Xindu Road, Yancheng 224001, China; ^3^Department of Pharmacy, Nanjing University of Chinese Medicine, Xianlin Road 138, Nanjing 210023, China

## Abstract

Puerarin is the main biologically active isoflavone in *Pueraria lobata* and has a wide range of biological activities. However, due to its poor water solubility and low oral bioavailability, its clinical applications are restricted. Compared with puerarin, the *Pueraria lobata* extract (PLE) has better water solubility, lower toxicity, and less side effects. In this study, the pharmacokinetics of orally administered puerarin (100 mg/kg) and PLE (763 mg/kg, equivalent to 100.0 mg/kg of puerarin) to rats was investigated by the UHPLC-MS/MS method. Results showed that when the rats were administered PLE, the area under the concentration-time curve from zero to infinity (*AUC*_*0-inf*_) dramatically increased from 219.83 ± 64.37 *μ*g h/L to 462.62 ± 51.74 *μ*g h/L (*p* < 0.01). The elimination half-time (*t*_*1/2*_) also increased from 1.60 ± 0.38 h to 12.04 ± 5.10 h (*p* < 0.01). The maximum concentration (*C*_max_) of puerarin decreased from 101.64 ± 41.82 ng/mL to 48.64 ± 21.47 ng/mL (*p* < 0.01), and time to reach the maximum plasma concentration (*T*_max_) of puerarin decreased from 1.46 ± 1.08 h to 0.54 ± 0.30 h (*p* < 0.01). Results indicated that the pharmacokinetics of puerarin in *Pueraria lobata* may be dramatically different from pure puerarin in the plasma of rat, and oral bioavailability of puerarin may be increased when PLE was administrated to rats.

## 1. Introduction

**Figure 1:**
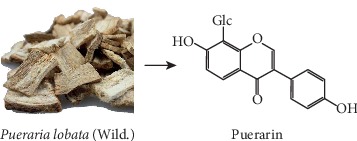
*Pueraria lobata* (Gegen) and the structure of PUR.

*Pueraria lobata* (Wild.) Ohwi (kudzu vine root, known as Gegen in China, Figure 1) is a traditional Chinese medicine (TCM) that has been used in clinical practice for thousands of years. Gegen grows widely in China, Korea, and Japan and used as a dietary supplement/functional food as well as herbal medicine. Modern research found that Gegen has a wide range of biological activities, such as antioxidant [1], anti-inflammatory [2], antidiabetic [3, 4], cardiovascular [4], antimutagenic [5], neuroprotective [6, 7], and antiestrogenic [8]activities.

Puerarin (daidzein-8-C-glucoside, PUR, Figure 1) is the major bioactive isoflavone in *Pueraria lobata* [9]. In China and other Asian countries, it is commonly used to prevent and treat cardiovascular and cerebrovascular diseases [10], including arteriosclerosis, hypertension, heart failure, and myocardial ischemia. PUR has also been reported to have protective effects of inflammation [11], hyperlipidemia [12], and oxidative stress [13]; it was even reported to improve vascular insulin resistance [14]. However, due to its poor water solubility and low oral bioavailability [15], the formulation of PUR is currently in an injection dosage form, while the injection has poor clinical compliance with patients, occasionally causing adverse drug reactions (ADRs). In addition, due to the frequent occurrence of side effects of TCM injection, China has restricted the use of TCM injection in recent years. These reasons limit its clinical application. In addition, due to the short elimination half-life of PUR in the human body, frequent and high-dose intravenous administration may be required, which may lead to acute side effects such as hemolysis, acute renal failure, and anaphylactic shock [16]. Therefore, oral formulations that improve PUR absorption have been one of the research priorities [17–22]. Compared with PUR, PLE has better water solubility, lower toxicity, and less side effects, while the pharmacokinetics of PUR in rats has not been compared yet.

There are many ingredients in a single herbal medicine, and the coexistence of these ingredients may affect the pharmacokinetic parameters and pharmacological effects of the main active compounds. Therefore, the pharmacokinetic characteristics of herbal extracts and single compounds purified from herbal extracts should be studied and compared. The purpose of this study was to develop a simple, reliable, and sensitive UHPLC-MS/MS method to determine the concentration of PUR in rat plasma and to compare the pharmacokinetics of PUR in rats after oral administration of PLE and pure PUR.

## 2. Materials and Methods

### 2.1. Chemicals and Reagents

PUR (purity > 98%) and berberine hydrochloride (purity > 98%) were purchased from Chengdu Herbpurify Co. Ltd. (Beijing, China), and berberine hydrochloride is the internal standard (IS) used in UHPLC-MS/MS analysis. Gegen was purchased from Beijing Tong Ren Tang Co, Ltd. (Yancheng, China). Acetonitrile (HPLC grade) and formic acid used were purchased from Thermo Fisher Scientific Company (USA). Ultrapure water (18.2 MΩ) was prepared by a Milli-Q water purification system (Millipore, France).

### 2.2. Preparation of PLE

Pieces of Gegen 100.0 g were immersed in a 10-fold volume of ethanol-water (70 : 30, v/v) for 30 min and then heated under reflux twice for 1 h each. The crude extract was concentrated by rotary evaporation at 40°C and then dried at 60°C with the vacuum drying method. The content of PUR in PLE was detected by the HPLC-UV method. The result would be used to calculate the oral administration dose.

### 2.3. Determination of PUR in PLE [23]

The measurement was performed on a Shimadzu HPLC 2010 HPLC system (Shimadzu, JAP) equipped with a UV detector. A SinoChrom ODS-AP column (150 mm × 4.6 mm, 5 *μ*m) was used. The column temperature was set at 30°C, and separation was performed by gradient elution with methanol (*A*) and water (*B*, containing 0.2% acetic acid). The flowrate was maintained at 1.0 mL/min. The detection wavelength was set at 250 nm. The gradient program was as follows: 0−5 min, 20%−30% A; 5−12 min, 30%−40% A; 12−25 min, 40%–85% A; 25−30 min, 85%–20% A. The injection volume was set at 10 *μ*L for the whole samples.

The appropriate amount of PLE and PUR was precisely weighed and then diluted with methanol to 25 mL. After filtration through a 0.22 *μ*m filter membrane, 10 *μ*L of PLE, PUR, and a reference filtrate solution was injected into the HPLC system. The content of PUR in PLE was then calculated. The result showed that the PUR content was 13.1% in PLE.

### 2.4. Animal Experiments

Male Wistar rats (250−270 g) were purchased from the SPF (Beijing) Biotechnology Co., Ltd. (Beijing, China). The rats were then housed in an animal room to adapt to the environment for a week at standard temperature (23−28°C), humidity (50−65%), free diet, and tap water. Rats were fasted for 12 h before pharmacokinetic experiments and allowed free access to water and sugar during sample collection. Both PUR and PLE were dispersed in a 0.5% carboxymethyl cellulose solution and then sonicated to obtain a homogeneous suspension. A whole blood sample was collected from the infraorbital vein and immediately centrifuged at 3,500 rpm for 10 minutes at 4°C to obtain plasma. Plasma was stored at −20°C for later analysis. The experimental protocol was approved by the Animal Experiment Ethics Review Committee of Jiangsu Vocational College of Medicine.

### 2.5. Instrumentation and Analytical Conditions [24]

Chromatographic separation was performed using a Waters Acquity UHPLC system with an Acquity HSS T3 column (1.8 *μ*m, 2.1 × 50 mm, Waters, Milford, MA). Mobile phase A consists of 0.1% aqueous formic acid solution, and mobile phase B consists of 0.1% formic acid acetonitrile. The gradient elution was as follows: 0 to 0.5 min, a linear gradient from 10 to 15% B; 0.5 to 2 min, 15 to 40% B; 2 to 4 min, 40 to 80% B; 4.1 to 5 min, 80 to 99% B; 5.1 to 7 min, a linear gradient back to 10% B. The flow rate was 0.3 mL/min. The column temperature was maintained at 40 °C, and the sample storage compartment was maintained at 4°C. Under these conditions, PUR and berberine hydrochloride were eluted at 2.0 and 3.2 minutes, respectively.

**Figure 2:**
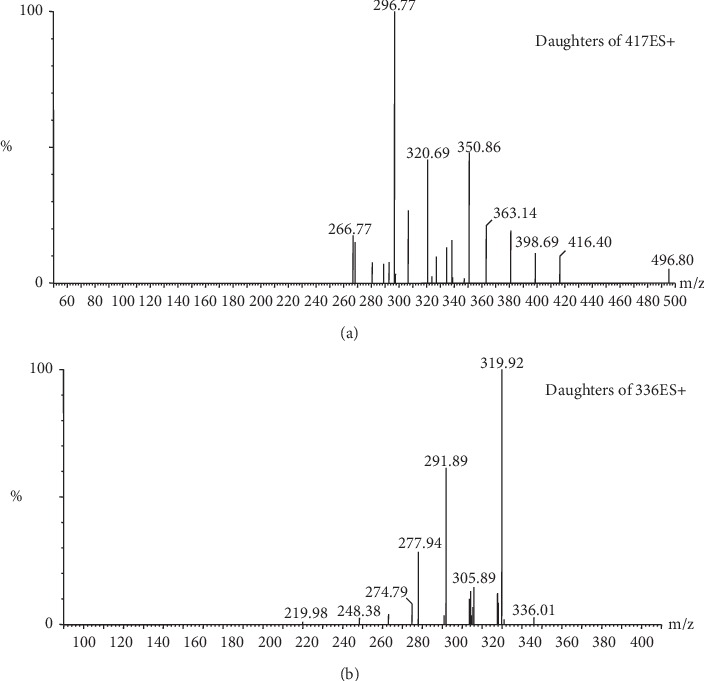
Mass fragment spectra of PUR (a) and berberine hydrochloride (b).

Mass spectrometric detection was performed on a Waters TQD tandem mass spectrometer equipped with an electrospray ionization (ESI) source. The mass spectrometer was operated in multiple reaction monitoring (MRM) positive-ion mode and was quantified using the following transitions: *m/z* 417.1 > 297.0 for PUR and *m/z* 336.0 > 320.0 for berberine hydrochloride, and the mass fragment spectrum of PUR and berberine hydrochloride is shown in Figure 2. For all transitions, the cone voltage of 35 V and a collision energy of 30 eV for both PUR and berberine hydrochloride were used, as shown in Table 1. The capillary voltage was set at 2.8 kV, and the source temperature was maintained at 500°C with a gas flow rate of 850 L/h. Argon is used as the collision gas. The cone gas flow was set at 50 L/h. Data acquisition were performed with MassLynx^TM^ software version 4.1, and quantitation was performed using TargetLynx^TM^ (Waters, Milford, MA, USA).

### 2.6. Preparation of Standard and Quality Control (QC) Samples

The stock solutions of PUR at a concentration of 200 *μ*g/mL were prepared with methanol. A series of working solutions (4000, 1000, 400, 200, 100, and 40 ng/mL) were then prepared by diluting the stock solution with methanol. A 100 *μ*g/mL IS stock solution was also prepared with methanol. A working solution of IS was prepared at a concentration of 100 ng/mL by diluting the stock solution with methanol. A standard calibration curve was prepared by adding 20 *μ*L of the abovementioned PUR working solution and 20 *μ*L of the IS working solution to 100 *μ*L of blank plasma. The final plasma concentrations of PUR were in the concentration ranges of 800, 200, 80, 40, 20, and 8 ng/mL. QC samples of low, medium, and high concentrations (8, 80, and 720 ng/mL) of PUR were also prepared following the same procedure as described above. All solutions were kept at 4°C until use.

### 2.7. Preparation of Plasma Samples

Rat plasma sample (100 *μ*L) and IS solution (20 *μ*L, 100 ng/mL) were added to a 1.5 mL Eppendorf tube and vortex-mixed for 30 s, followed by adding 500 *μ*L of acetonitrile to precipitate protein. The mixture was then vortex-mixed for 2 min and centrifuged at 10,000 rpm for 10 min. The supernatant was evaporated to dryness under a stream of nitrogen gas. The obtained residue was reconstituted with methanol-water (80 : 20, v/v), then vortex-mixed for 3min and centrifuged at 13,000 rpm for 10 min. Finally, a 2 *μ*L sample of the supernatant was injected into the UHPLC-MS/MS system for analysis.

### 2.8. Method Validation [24]

Method validation was carried out according to the FDA bioanalytical method validation guidance (2001). The blank plasma sample of rats was used to investigate the potential interference of endogenous components. Chromatograms of blank plasma, plasma spiked with analytes and IS, and plasma samples after oral administration of PUR suspension were compared.

Linearity was determined by plotting the peak-area ratio of PUR to IS versus its concentration. Linearity was evaluated by weighted least squares linear regression (weighting factor, 1/*x*^2^). The lower limit of quantification (LLOQ) was measured based on at least 10 times of signal-to-noise ratio.

Accuracy and precision were assessed by measuring QC samples at three concentration levels (8, 80, and 720 ng/mL) over three verification days and five replicates each. Precision was expressed as relative standard deviation (RSD), and accuracy was expressed as relative error (RE). The intraday (in one day) and interday (in three successive days) precision was required to be less than 15%, and the accuracy was also required to be within ± 15%. The LLOQ precision required no more than 20% and accuracy within ± 20%.

Extraction recovery was evaluated on the PUR of three concentration levels of QC samples and the IS at the concentration of 100 ng/mL, and each sample was repeated three times. It was determined by comparing the peak area of extracted plasma (prespiked) standard QC samples to those of postspiked standards at equivalent concentration. The Matrix effects were evaluated by comparing the peak areas of the PUR in the postextraction added samples with those of the pure standard solutions.

The stability of the PUR in rat plasma was investigated by analyzing QC samples under four different storage conditions, including three freeze-thaw cycles, room temperature 23−28°C for 24 h, −20°C for 30 days, and postextraction stability after the extracted samples being stored in the auto sampler at the temperature 4°C for 24 h. If the accuracy deviation was within ± 15%, the PUR was considered stable.

### 2.9. Application of the Assay to Pharmacokinetic Studies

The validated method was applied to determine the plasma concentration of PUR in rats after single oral administration of PLE at a dose of 763 mg/kg (equivalent to 100.0 mg/kg of PUR, 1 mL/100g BW) or administration of PUR at a dose of 100 mg/kg (1 mL/100g BW). Twelve male Wistar rats were randomly divided into two groups of six rats each and fasted for 12 h before the experiment. The whole blood samples were collected in heparinized tubes through the suborbital vein of rat at 0.25, 0.5, 0.75, 1, 1.5, 2, 3, 4, 6, 8, 12, 24, 36, and 48 h after gavage administration of drugs. The blood samples were immediately centrifuged at 3,500 rpm for 10 min to obtain plasma. Plasma was stored at −20°C until analysis.

### 2.10. Data Analysis

Pharmacokinetic parameters were evaluated by DAS software (version 2.0, Chinese Pharmacological Society, Shanghai, China), including the area under the plasma concentration-time curve (*AUC*_*0–t*_), the area under the plasma concentration-time curve from zero to infinity (*AUC*_*0–inf*_), time to reach the maximum plasma concentration (*T*_max_), elimination half-time (*t*_*1/2*_), the maximum plasma concentration (*C*_max_), mean residence time (*MRT*_*0-t*_), clearance (*CLz/F*), and apparent volume of distribution (*Vz/F*). Data were expressed in mean and standard deviation (SD) of each group.

A paired *t*-test was used to analyze the significance of the differences between the two groups. The value was considered statistically significant if *p* < 0.05 (SPSS 18.0 software, SPSS Inc., Chicago, IL, USA).

## 3. Results and Discussion

### 3.1. Optimization of Experiment

After oral administration of pure PUR and PLE to rats, the PK parameters of PUR in rat plasma were compared by the UHPLC-MS/MS method. In the experiment, a 50 mm UHPLC column was selected. Compared with a 100 mm column, it could significantly save the separation time and almost still maintain a good separation effect. The IS should be carefully selected before the experiment. It must be stable and cannot be a potential metabolite of any component in PLE. In this study, daidzein, baicalin, irisolidone, and berberine hydrochloride were tried. Daidzein and irisolidone may be potential metabolites of PLE. The baicalin structure was unstable in this study. Berberine hydrochloride with a stable structure and good mass spectrum peak shape was selected as IS.

### 3.2. Method Validation

#### 3.2.1. Selectivity

**Figure 3:**
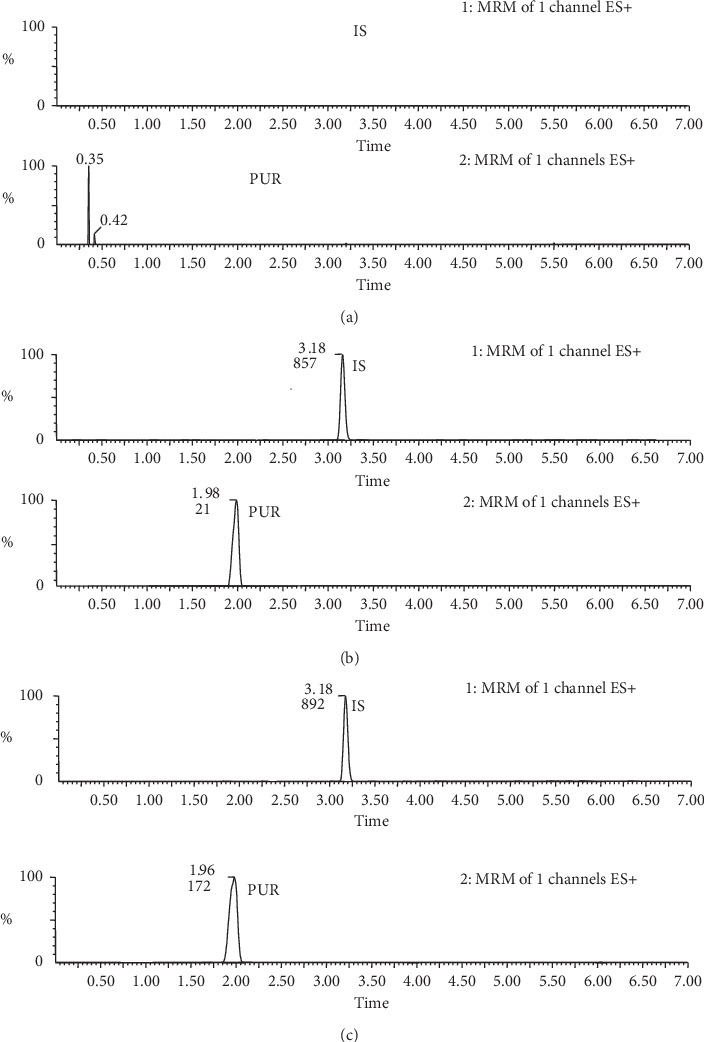
Representative chromatograms of blank plasma (a); blank plasma spiked with PUR at LLOQ level and IS (b); plasma sample after oral administration of PLE at 0.25 h (c).

Extracted ion chromatography images (EIC) of PUR and IS from rat plasma samples and blank rat plasma samples are shown in Figure 3. Results showed that there was no endogenous peak interference to the EIC of the PUR. No interfering peaks for PUR and IS were observed in plasma samples, as the exclusive detection mode of the ion pair of MRM resulted in less endogenous interference.

#### 3.2.2. Linearity and Sensitivity

The typical equation of the calibration curve of PUR was described as follows: *y* = 0.909x − 2.648, *r*^2^ = 0.9991, where *y* represents the peak-area ratio of analytes to IS and *x* represents the plasma concentration of PUR. The correlation coefficient (*r*^2^) exceeded 0.99, indicating a good linearity in the tested concentration range. The lower limit of quantification (LLOQ) of PUR was defined as half of the lowest concentration on the calibration curve with 4 ng/mL.

#### 3.2.3. Accuracy and Precision

As shown in Table 2, the precision and accuracy of the method were determined by analyzing the QC samples and LLOQ samples (*n* = 5). All values were within acceptable ranges, and the method was considered accurate and precise.

#### 3.2.4. Recovery and Matrix Effect

Results are shown in Table 3. Extraction recovery of PUR ranged from 85.6% to 93.4% and IS at 106.4%. The matrix effect of PUR ranged from 108.8% to 113.2% and IS at 95.3%. All values were within the allowed range.

#### 3.2.5. Stability

PUR was stable after three complete freeze/thaw cycles (−20°C to 25°C), long-term sample storage (−20°C for 30 days), benchtop (23°C to 28°C for 24 h), and postextraction (4°C for 24 h). The stability results presented in Table 4 indicate that the analytes were stable under four tested conditions.

### 3.3. Application of Pharmacokinetic Study in Wistar Rats

**Figure 4:**
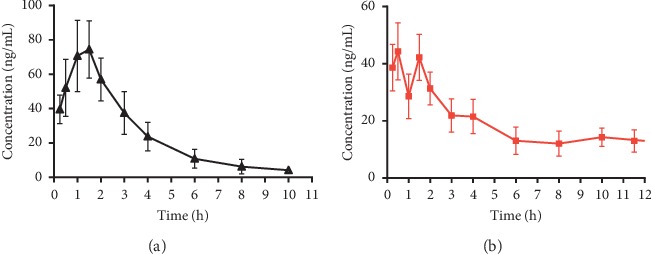
Plasma concentration-time curves for PUR in rats after oral administration of (100 mg/kg PUR, (a)) and (763 mg/kg PLE, (b)).

The validated UHPLC-MS/MS method was used to quantify plasma concentrations of PUR after oral gavage administration of PUR at a dose of 100 mg/kg or PLE at a dose of 763 mg/kg to rats. Their mean plasma concentration-time profiles are shown in Figure 4(a) and Figure 4(b), respectively, and the pharmacokinetic parameters, such as *C*_max_, *T*_max_, *t*_*1/2*_, *AUC*_*0–inf*_, *AUC*_*0–t*_, *MRT*_*0-t*_, *CLz/F*, and *Vz/F* are summarized in Table 5.

There was no double-peak phenomenon on the plasma concentration-time curve of PUR when pure PUR was administrated to rats, as shown in Figure 4 and other studies [25, 26], while a clear double-peak phenomenon was observed after oral administration of PLE. The double-peak phenomenon could not simply be interpreted by entering the enterohepatic circulation.

As shown in Table 5, *AUC*_*0-t*_*and AUC*_*0-inf*_ of PUR after oral administration of PLE were 406.30 ± 53.68 *μ*g h/L and 462.62 ± 51.74 *μ*g h/L, respectively, which were significantly higher than those of the PUR group with 213.09 ± 61.91 *μ*g h/L and 219.83 ± 64.37 *μ*g h/L, respectively (*P* < 0.01). The *t*_*1/2*_ value of PUR in the PLE group was prolonged from 1.60 ± 0.38 h to 12.04 ± 5.10 h (*P* < 0.01), and the *CLz/F* was also notably reduced from 508.66 ± 202.54 to 217.30 ± 25.04 (*P* < 0.01). However, the *C*_max_ value of PUR was 48.64 ± 21.47 *μ*g/L, and the *T*_max_ was 0.54 ± 0.30 h, which were lower than those of the PUR group (*P* < 0.01). Results showed that oral administration of PLE delayed *T*_max_ of PUR and reduced *C*_max_; however, the blood concentration of PUR could maintain at a relative suitable high level for a long time, which was beneficial to the drug-forming properties. The results showed that the pharmacokinetics of PUR after oral administration of PLE was significantly different from the administration of PUR in rat plasma. When PLE was administered to rats, the oral bioavailability of PUR increased. Studies have found that PUR could be biotransformed to puerarin-7-O-glucuronide and puerarin-4′ -O-glucuronide in vivo, which resulted in a lower oral bioavailability of PUR [27, 28]. A common phenomenon happens to most TCM monomer components. For example, after oral administration of kakkalide, many related metabolites were detected in rat plasma, and kakkalide only accounted for a small part of the content of metabolites [29]. Many metabolites were also detected in the plasma of rat after oral administration of irisolidone, especially glucuronide metabolites and sulfate metabolites [23].

The HPLC-UV method was used to detect the PUR content in PLE. The amount of PUR in PLE administered to rats was the same as the PUR of 100 mg/kg BW. However, only 13.1% PUR was detected in PLE, which would increase the oral dosage of PLE. And, there exist many isoflavones with similar structure in PLE, which might be converted to PUR in vivo. This could be one of the reasons for the increased bioavailability of PUR after oral administration of PLE [24]. Another reason was drug interactions between PUR and other ingredients in PLE. For example, compared with the control group, both glycyrrhizin and astragaloside IV can significantly reduce the *T*_max_, *C*_max_, and *AUC*_*0-t*_ of PUR, which indicates that the drug interaction between glycyrrhizin and astragaloside IV with PUR cannot be ignored when they were coadministered [25, 26]. Studies have found that after oral administration of the *Iris tectorum* Maxim extract, the bioavailability of tectorigenin in rat plasma has also increased significantly compared with administration of pure tectoridin [24], and we obtained similar results in this study. As we all know, oral drugs are mainly absorbed in the small intestine and there are a large number of metabolic enzymes and bacteria in the intestine, which catalyzes the drug's metabolic response and ultimately affects drug absorption. Various transport proteins in the small intestinal epithelial cells can promote or inhibit drug absorption [30]. Many kinds of TCM compounds have been shown to bind to P-gp proteins. MRP2 protein can also reduce the absorption of oral drugs by combining with drug metabolites. Studies have found that after added verapamil (P-gp inhibitor) and probenecid (MRP2 inhibitor), the intestinal absorption of PUR increased by 2.7 and 2.3 times, respectively, indicating that PUR may be a substrate for P-gp and MRP2 proteins. There exist multiple similar isoflavone components in PLE, which might become substrates for P-gp and MRP2 proteins, as well as substrates for various metabolic enzymes and bacteria in the rat intestine, thus competing with PUR and reducing the response of PUR.

## 4. Conclusion

In summary, this was the first report to compare the plasma pharmacokinetics of PUR in rats after oral administration of PUR and PLE using a simple, rapid, and sensitive UHPLC-MS/MS method. Oral bioavailability of PUR was dramatically increased when PLE was administrated to rats. These results provide useful information for clinical usage of PUR and *Pueraria lobata*, and the traditional herbal medicine has its advantages over its pure ingredient after oral administration although the dosage might be higher than the monomer compound.

## Figures and Tables

**Table 1:** Precursor/production pairs and parameters for MRM of PUR and IS.

Analyte	Ionization mode	MRM transitions	Cone voltage (v)	Collision energy (ev)
PUR	Positive	417.1 > 297.0	35	30
IS	Positive	336.0 > 320.0	35	30

**Table 2:** Precision and accuracy of PUR by the UHPLC-MS/MS method in rat plasma (*n* = 5).

Marker compounds	Concentration (ng/mL)		RSD (%)		RE (%)
	Added	Found	Intraday	Interday	
PUR	8.0	7.1	13.8	15.3	−11.3
	80.0	75.0	8.8	6.7	−6.3
	720.0	647.0	6.6	7.8	−10.1

LLOQ	4.0	4.7	18.9	15.8	17.5

**Table 3:** Recovery and matrix effects of PUR and IS in rat plasma by the UHPLC-MS/MS method (*n* = 5).

Marker compounds	Concentration (ng/mL)	Recovery (%)	RSD (%)	Matrix effect (%)	RSD (%)
PUR	8.0	85.6	14.5	113.2	7.4
	80.0	95.1	9.3	108.8	8.7
	720.0	93.4	10.9	112.5	5.9

IS	100.0	106.4	8.7	95.3	9.5

**Table 4:** Stability of PUR in rat plasma by the UHPLC-MS/MS method (*n* = 3).

Marker compounds	Concentration (ng/mL)	Stability (RE, %)			
		Freeze-thaw (3 cycles)	23°C–28°C/24 h	−20°C/30 day	Postextraction
PUR	8.0	9.3	11.5	11.4	−7.7
	80.0	5.6	6.5	6.2	−6.3
	720.0	−5.1	8.7	−4.7	5.9

**Table 5:** Pharmacokinetic parameters of PUR in rat plasma after oral administration of PUR at a dose of 100 mg/kg and PLE at a dose of 763 mg/kg. (*n* = 6, mean ± SD).

Parameters	Units	PUR	PLE
*AUC*_*0-t*_	*μ*g·h/L	213.09 ± 61.91	406.30 ± 53.68^*∗∗*^
*AUC*_*0-inf*_	*μ*g·h/L	219.83 ± 64.37	462.62 ± 51.74^*∗∗*^
*MRT*_*0-t*_	h	2.22 ± 0.46	11.28 ± 0.79^*∗*^
*t*_*1/2*_	h	1.60 ± 0.38	12.04 ± 5.10^*∗∗*^
*T*_max_	h	1.46 ± 1.08	0.54 ± 0.30^*∗∗*^
*CLz/F*	L/h/kg	508.66 ± 202.54	217.30 ± 25.04^*∗∗*^
*Vz/F*	L/kg	1198.32 ± 403.31	3738.75 ± 1436.17^*∗*^
*C*_max_	*μ*g/L	101.64 ± 41.82	48.64 ± 21.47^*∗∗*^

^*∗*^ *p* < 0.05; ^*∗∗*^*p* < 0.01, compared with the PUR group.

## Data Availability

All data contained in the manuscript will be made available from the corresponding author upon reasonable request.

## Additional Points

*Highlights.* A novel UPLC-MS/MS method was established for PK of puerarin. A comparative PK was performed between the herb extract and the pure compound. The coexisting components in the herb extract promoted the absorption of the pure compound. The potential interaction between the ingredients of the herb extract may exist.
